# Bilateral renal infarction following atrial fibrillation and thromboembolism and presenting as acute abdominal pain: a case report

**DOI:** 10.1186/1752-1947-6-153

**Published:** 2012-06-13

**Authors:** Khaireddine Bouassida, Wissem Hmida, Amira Zairi, Adnen Hidoussi, Mehdi Jaidane, Adel Slama, Nebil Ben Sorba, Khaled Hani, Ali Faouzi Mosbah

**Affiliations:** 1 Department of Urology, Hospital of Sahloul, Sousse, Tunisia; 2 Laboratory of Biochemistry, Faculty of Medicine, Sousse, Tunisia

**Keywords:** Renal infarct, Renal failure, Acute abdominal ache, Atrial fibrillation arrhythmia, Cortical rim sign.

## Abstract

**Introduction:**

Renal infarct is rare and often misdiagnosed because the symptoms are misleading. The mechanisms are various, mainly thrombotic and embolic.

**Case presentation:**

In this review, we report the case of a 61-year-old Tunisian woman presented to the emergency unit with a 4-hour history of abdominal pain diffused at both flanks, ultrasounds was performed to remove a surgical emergency, showed a peri-renal fluid collection with heterogeneous parenchyma.

We followed by a CT scan, which confirmed the diagnosis of renal infarct. The patient was treated by heparin at a curative dose, and the outcome was favorable.

**Conclusion:**

Diagnosis is difficult and should be considered in patients with inexplicable flank or abdominal pain and with risk factors to this disease. Our purpose is to raise clinician’s awareness for this condition so that they will be more likely to diagnose it. This will facilitate prompt diagnosis and treatment.

A review of the literature was performed and the case is discussed in the context of the current knowledge of this condition.

## Introduction

Detection of acute renal infarction is often delayed or missed due to the rareness of this disease. The mechanisms are various, mainly thrombotic and embolic [1]. The bilateral occurrence in the context of atrial fibrillation responsible for acute renal failure is exceptional and to our knowledge, no cases have previously been reported. The diagnosis should be considered in inexplicable acute abdominal pain sometimes associated with hematuria especially with patients at risk for embolic events (atrial fibrillation, endocarditis, atherosclerotic aorta) [1,2]. We report the case of bilateral renal infarction associated with acute renal failure due to an embolus from the heart caused by atrial fibrillation.

## Case presentation

We report the case of a 61-year-old Tunisian woman presented to the emergency unit with a 4-hour history of abdominal pain, diffused at both flanks without hematuria. Her medical history was significant for diabetes, hypertension and atrial fibrillation, besides her surgical history indicates left pneumectomy for pulmonary tuberculosis. Her medications consisted of aspirin, metformin, furosemide, and metoprolol. Physical examination showed vital signs as follows: body temperature of 37.2°C, blood pressure of 150/85 mmHg, heart rate of 133 beats per minute, respiratory rate of 20 breaths per minute; the abdomen was soft with slight tenderness in the renal angles. The remainder of the examination was normal.

An electrocardiogram showed atrial fibrillation. A biological analysis showed high serum creatinine levels (205μmol/L); her aminotransferases were twice normal the normal levels and her lactate dehydrogenase (LDH) level had increased to (8,636IU/L) with high leukocytosis at 15,000 cells/mm^3^. A urine analysis showed no hematuria or infection. A serum analysis done one month previously showed that her creatinine level was normal (93μmol/L). Nephrolithiasis and mesenteric ischemia were strongly suspected to be the causes for this acute pain.

**Figure 1:**
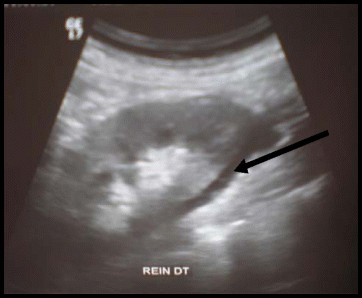
Renal ultrasnography: a peri-renal fluid collection with heterogeneous parenchyma.

**Figure 2:**
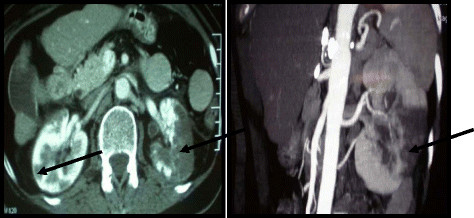
Axial and Coronal enhanced CT angiography: parenchymal perfusion defects of both kidneys related to segmental distal arterial lesions.

Ultrasounds of the abdomen were performed and showed peri-renal fluid collection with heterogeneous parenchyma (Figure 1). Contrast-enhanced helical computed tomography (CT) images showed: a thrombus in the left auricle, an increase in kidney’s size besides to perirenal edema and multiple parenchymal perfusion defects of both kidneys that are related to segmental distal arterial lesions which suggest arterial thromboembolic disease. Aorta and renal arteries were permeable with no atherosclerotic plaque; the renal vein and inferior vena cava were both less permeable. Figure 2.

**Figure 3:**
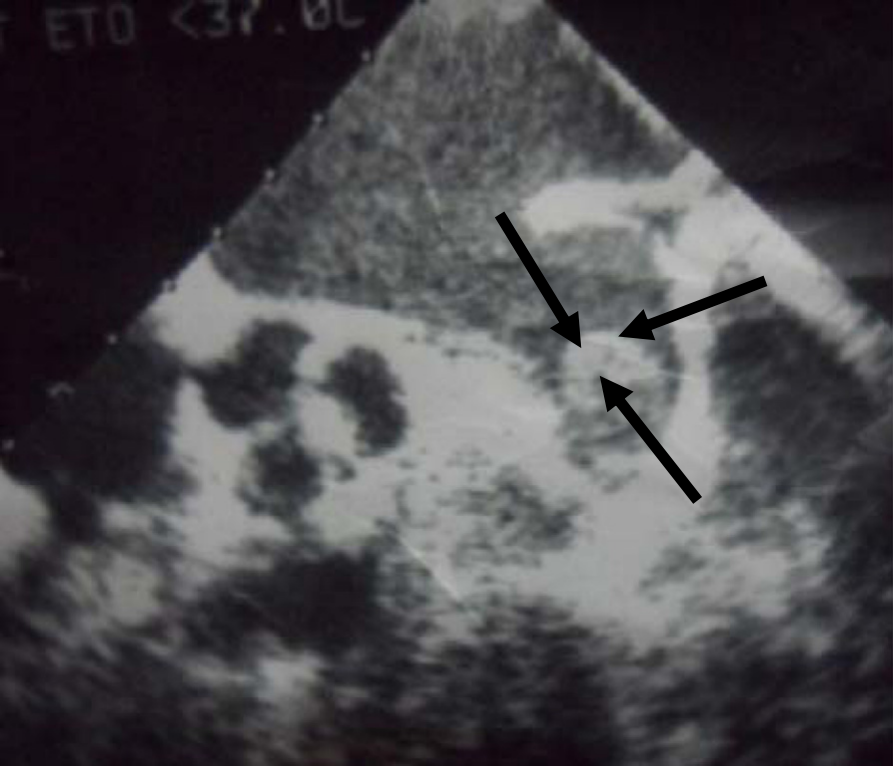
Transesophageal echocardiography: presence of a thrombus in the left atrium.

Subsequent transesophageal echocardiography confirmed the presence of a thrombus in the left atrium without valvular vegetations Figure 3.

We concluded that it was a bilateral renal infarction of embolic origin caused by migration of an embolus from the left atrium. In addition to analgesic, continuous IV infusion of heparin was administered at the curative dose of 18 units/kg/hour and the outcome was favorable: the abdominal pain disappeared besides to a decrease of serum creatinine to (115 μmol/ l) after 3 days. One month later, the creatinine levels declined to 95 μmol/ l so we also concluded that acute renal failure was due to the infarction.

## Discussion

The renal infarction is a rare disease, difficult to be diagnosed because the symptoms are misleading, in addition to renal calculi, many other disorders such as intestinal diseases (including mesenteric ischemia), spinal disease, genital disease, muscle inflammation, myocardial infarction or ischemia, also show similar symptoms. Renal infarction, therefore, is often misdiagnosed [1].

Infarction of kidney can result from blockage of arterial or venous drainage. Problems related to arterial supply is far more common than the venous abnormalities [2]. The incidence is higher in patients with atherosclerosis, kidney damage (nephritic syndrome, glomerulonephritis), fibromuscular disease, aneurysms and dissections of the renal artery) [3].

Bilateral infarction was reported to be found in dissecting aneurysms of the aorta [4] with septic emboli from endocarditis, lupus, vasculitis, sickle cell disease [1] or with fibromuscular dysplasia of the renal arteries [5]. Renal infarct has a state of alert since cerebral and peripheral embolism can occur at any time [6].

Renal infarct affects elderly patients, with an average age of 67 with no significant gender predominance or right or left kidney predominance [7]. Patients generally complain of flank pain and/or upper abdominal pain which are often associated with nausea and vomiting. Occasionally, patients may have hematuria [8], fever is also common and was present in about half of the reported cases. Blood pressure may be acutely elevated, and this is presumed to be through a renin-mediated mechanism. Signs of extrarenal embolism may also be present [1]. In contrast, renal infarct may be completely asymptomatic and then diagnosed incidentally as a result of abdominal CT examination [8].

In terms of laboratory tests, several serum markers have been suggested as indicative of renal infarction such as elevations in alkaline phosphatase, fibrinogen, C-reactive protein (CRP), and aspartate. These markers are inconsistently elevated [9,10]. Serum LDH appears to be the most sensitive marker as it was elevated in most of the cases described [10,11]. The white blood cell count is often high [7] and the significant increase of LDH in our case could be attributed to bilateral disease. Renal function can be affected in solitary kidney or after bilateral infraction.

Mechanisms of renal infarction are multiple and varied. They may be embolic, typically caused by blood or cholesterol clots occluding the renal artery or branch vessels. This is the most common cause and is often of cardiac origin, such as atrial fibrillation, as in the case of our patient, myocardial infarction with left ventricular thrombus, mitral stenosis, atrial myxoma or infective endocarditis [3,4]. Infarction can be caused by thrombosis, such as the occlusion of a renal artery by a thrombus, aneurysm of the renal artery, thromboangiitis obliterans or thrombocytopenic purpura [3]. Traumatic laceration of a renal artery (grade III lesion) or a segmental branch (grade II lesion) [3] may also result in renal infarction. Some narcotics, such as marijuana or cocaine have also been involved [4]. Finally, the renal infarction may be idiopathic: a case of renal infarction was recently reported without any obvious etiology [4].

Imaging is the key to diagnose renal artery occlusion. Although noncontrast-enhanced CT is not required, yet it often provides us with information to exclude other diagnoses, such as renal calculi, hemorrhagic cyst, or renal or perirenal hematoma [8].

The CT scan has a good sensitivity about 85% mainly with reconstruction thickness of 5 mm [10]. The radiological features on CT are represented by a well-known sign: the "cortex corticis" or "cortical rim sign" [12]. This sign is most commonly found with renal artery obstruction from thrombosis, embolus or dissection. During contrast, enhanced CT or MR imaging, a 1- to 3-mm rim of subcapsular enhancement paralleling the renal margin, can be detected as a result of preserved perfusion of the outer renal cortex by capsular perforating vessels. The finding may be partial or total depending on the level of vascular occlusion, and there may be an abrupt termination of contrast material in the renal artery referred to as the arterial cut-off-sign [8]. This sign is pathognomonic, but it is often absent during the first six hours following the infarct and is infrequent (present in about 50% of cases), especially in old and chronic infarcts [2].

A recent CT sign was also highlighted and described as a ‘flip-flop’ enhancement which is a delayed enhancement of the initial hypo attenuations [2]. This sign is due to extravasation of contrast material in areas of ischemia caused by disruption of the glomerular membrane. This sign is suggesting of ischemia rather than necrosis and may be seen in inflammatory states [2].

The main differential diagnosis for renal infarcts are acute pyelonephritis but the subcapsular region does not enhance, and abnormal perirenal fats are often more important. Other diagnoses to be considered are lymphoma or metastases [1,9].

If the patient is at risk for embolic events and has an elevated serum LDH, it is recommended to undergo renal isotope exam (scintography) or renal angiography if the contrast-enhanced CT is negative [7].

Therapeutic guidelines for the treatment of renal embolism have not been established. Prompt recognition of acute occlusion of the renal artery is important since thrombolysis, anticoagulation, or embolectomy may minimize the loss in renal function [9].In 1973, Moyer and his workers compared the results of surgical and medical management for unilateral renal embolism and found conservative therapy to be favored ([13]).Because of the infrequent nature of this disease, there are only recommendations based on the consensus in the literature, no large prospective studies have evaluated the optimal treatment modalities [11].

The standard treatment strategy includes anticoagulation with or without thrombolysis [1].

A heparin bolus followed by initiation of warfarin sodium herapy is recommended [7]. The dose of initial heparin, the target international normalized ratio (INR) and the length of warfarin sodium therapy have not been defined [1,7]. Perfusion can also be reestablished through thrombolysis or surgical revascularization [10,13]. There is no consensus regarding this issue, and the exact role of intra-arterial thrombolysis is yet to be determined. Open surgery is not recommended other than in the case of trauma. In one case report, embolectomy had been performed 30 hours after complete bilateral occlusion with complete resolution of kidney failure [7]. Local thrombolysis or thrombectomy with minimally invasive percutaneous endovascular therapy for acute occlusions of the main renal artery or significant branch can be considered [14]. Patients with prior hypertension or with new-onset hypertension from the infarct should be treated with antihypertensive therapy [12] because of the insult to the kidney or persistent hypertension, but a majority return to their baseline renal function with no permanent hypertension [12]. A small percentage will need dialysis, 8% in one case series [7].

Despite the uncertainty regarding management, the renal outcome remains favorable. Many patients do develop some degree of renal insufficiency during the acute episode. The death rate among these patients is typically caused by recurrent embolic disease or heart disease rather than renal complications [10] Table 1.

**Table 1:** Outcomes in Renal Artery Embolism [6,10,15]

**Outcome**		**Patients’ percentages**
Renal function	Normal renal function	57.7
	Mild renal impairment (creatinine <2mg/dL)	16.7
	Moderate renal impairment (creatinine >2mg/dL)	15.4
	Severe renal impairment	10.2
Death at one year		14.3
Associated conditions	Embolic disease	50.0
	Myocardial infarction	25.0
	Sepsis	25.0

Because of the lack of prospective study data, it is unknown what the benefit of continuing anticoagulation would be. However it is recommended to continue indefinitely in the setting of atrial fibrillation [10].

## Conclusion

Bilateral renal infarction is a rare condition. No cases of bilateral renal infarction following an atrial fibrillation have previously been reported to our knowledge. It may involve the functional outcome of both kidneys and has an alert for this patient because renal infarct is threatened by the occurrence of other thromboembolic events in other various areas, including the brain [14]. Diagnosis is difficult and should be considered when presented with symptoms of acute abdominal pain, renal colic or in a particular context. The use of CT remains quite valuable to confirm the diagnosis. The treatment must be at an early stage and based on the use of anticoagulants [15]. Our purpose is to increase awareness of this condition so that clinicians will consider diagnosing it. This will facilitate prompt diagnosis and treatment.

### Patient consent section

Written informed consent was obtained from the patient for publication of this case report and accompanying images. A copy of the written consent is available for review by the Editor-in-Chief of this journal.

## Competing interest

I declare that I have no competing interests. The authors declare that they have no competing interests

## Authors’ contributions

KB and WB analyzed and interpreted the patient data. AH and FM performed the radiological examination. AZ and MJ were the major contributors in writing the manuscript. KH and AFM supervised the hole work. All authors read and approved the final manuscript.
